# Clinician- and Patient-Directed Communication Strategies for Patients With Cancer at High Mortality Risk: A Cluster Randomized Trial

**DOI:** 10.1001/jamanetworkopen.2024.18639

**Published:** 2024-07-01

**Authors:** Samuel U. Takvorian, Peter Gabriel, E. Paul Wileyto, Daniel Blumenthal, Sharon Tejada, Alicia B. W. Clifton, David A. Asch, Alison M. Buttenheim, Katharine A. Rendle, Rachel C. Shelton, Krisda H. Chaiyachati, Oluwadamilola M. Fayanju, Susan Ware, Lynn M. Schuchter, Pallavi Kumar, Tasnim Salam, Adina Lieberman, Daniel Ragusano, Anna-Marika Bauer, Callie A. Scott, Lawrence N. Shulman, Robert Schnoll, Rinad S. Beidas, Justin E. Bekelman, Ravi B. Parikh

**Affiliations:** 1 Perelman School of Medicine, University of Pennsylvania, Philadelphia; 2 Wicked Saints Studios, Medford, Oregon; 3 School of Nursing, University of Pennsylvania, Philadelphia; 4 Department of Sociomedical Sciences, Mailman School of Public Health, Columbia University, New York, New York; 5 Verily Life Sciences, San Francisco, California; 6 New Jersey Department of Health Communicable Disease Service, Trenton, New Jersey; 7 School of Medicine, American University of the Caribbean, Cupecoy, Sint Maarten; 8 Critical Path Institute, Tucson, Arizona; 9 Cohere Health, Ann Arbor, Michigan; 10 Department of Medical Social Sciences, Feinberg School of Medicine, Northwestern University, Chicago, Illinois

## Abstract

**Importance:**

Serious illness conversations (SICs) that elicit patients’ values, goals, and care preferences reduce anxiety and depression and improve quality of life, but occur infrequently for patients with cancer. Behavioral economic implementation strategies (nudges) directed at clinicians and/or patients may increase SIC completion.

**Objective:**

To test the independent and combined effects of clinician and patient nudges on SIC completion.

**Design, Setting, and Participants:**

A 2 × 2 factorial, cluster randomized trial was conducted from September 7, 2021, to March 11, 2022, at oncology clinics across 4 hospitals and 6 community sites within a large academic health system in Pennsylvania and New Jersey among 163 medical and gynecologic oncology clinicians and 4450 patients with cancer at high risk of mortality (≥10% risk of 180-day mortality).

**Interventions:**

Clinician clusters and patients were independently randomized to receive usual care vs nudges, resulting in 4 arms: (1) active control, operating for 2 years prior to trial start, consisting of clinician text message reminders to complete SICs for patients at high mortality risk; (2) clinician nudge only, consisting of active control plus weekly peer comparisons of clinician-level SIC completion rates; (3) patient nudge only, consisting of active control plus a preclinic electronic communication designed to prime patients for SICs; and (4) combined clinician and patient nudges.

**Main Outcomes and Measures:**

The primary outcome was a documented SIC in the electronic health record within 6 months of a participant’s first clinic visit after randomization. Analysis was performed on an intent-to-treat basis at the patient level.

**Results:**

The study accrued 4450 patients (median age, 67 years [IQR, 59-75 years]; 2352 women [52.9%]) seen by 163 clinicians, randomized to active control (n = 1004), clinician nudge (n = 1179), patient nudge (n = 997), or combined nudges (n = 1270). Overall patient-level rates of 6-month SIC completion were 11.2% for the active control arm (112 of 1004), 11.5% for the clinician nudge arm (136 of 1179), 11.5% for the patient nudge arm (115 of 997), and 14.1% for the combined nudge arm (179 of 1270). Compared with active control, the combined nudges were associated with an increase in SIC rates (ratio of hazard ratios [rHR], 1.55 [95% CI, 1.00-2.40]; *P* = .049), whereas the clinician nudge (HR, 0.95 [95% CI, 0.64-1.41; *P* = .79) and patient nudge (HR, 0.99 [95% CI, 0.73-1.33]; *P* = .93) were not.

**Conclusions and Relevance:**

In this cluster randomized trial, nudges combining clinician peer comparisons with patient priming questionnaires were associated with a marginal increase in documented SICs compared with an active control. Combining clinician- and patient-directed nudges may help to promote SICs in routine cancer care.

**Trial Registration:**

ClinicalTrials.gov Identifier: NCT04867850

## Key Points

**Question:**

Can behavioral economic implementation strategies (“nudges”) for clinicians and/or patients improve rates of serious illness conversation (SIC) among patients with cancer at high risk of 180-day mortality?

**Findings:**

In this 2 × 2 factorial, cluster randomized clinical trial including 4450 patients with cancer across academic and community sites, combined clinician and patient nudges were associated with a marginal improvement in rates of SIC documentation (ratio of hazard ratios, 1.55) compared with an active control.

**Meaning:**

Combining clinician- and patient-directed nudges may help to promote SICs in routine cancer care.

## Teaser

This cluster randomized trial tests the independent and combined effects of clinician and patient behavioral economic implementation strategies (nudges) on completion of serious illness conversations that elicit patients’ values, goals, and care preferences.

## Introduction

Patients with cancer often experience distress, use unplanned acute care, and undergo unwanted medical interventions, especially at the end of life.^1,2,3,4,5,6^ Serious illness conversations (SICs) that elicit patients’ values, goals, and care preferences improve patient mood and quality of life^7,8,9,10,11,12^ and may reduce end-of-life health care utilization.^13^ Early SICs are evidence-based and recommended by national guidelines.^14,15,16^ However, most patients with cancer die without a documented SIC, a situation associated with unwarranted aggressive end-of-life care.^7^ Existing strategies to promote SICs have focused on clinician education and/or detailed patient readiness training, but have variably affected the timeliness and frequency of SICs (eTable 1 in Supplement 2).^11,12^ For example, prospective trials of clinician behavioral economic strategies (nudges) and patient readiness trainings have resulted in 2-fold to 4-fold increases in SICs,^17,18^ but trials of similar interventions in other contexts have had smaller effects.^19,20^

Nudges may help to implement evidence-based practices such as SICs by targeting mental heuristics that influence decision-making.^21,22^ Clinician-level factors that undermine SIC completion include avoidance, time constraints, inadequate training, fears of removing patients’ hope, and optimism bias (the belief that one’s own patient is unlikely to experience a negative outcome).^17,23,24,25^ Due to optimism bias, clinicians overestimate the life expectancy of patients with advanced cancer,^26,27,28^ potentially reinforcing similar biases among patients and contributing to delayed SICs, which may reinforce a social norm for both clinicians and patients that SICs are not appropriate until near the end of life.^16,29,30,31^ Such social norms are powerful behavioral determinants: individuals desire to conform to an approved behavior (an injunctive norm) and the behavior of others (a descriptive norm).^32^

Limited research has evaluated behaviorally informed implementation strategies designed to harness these norms to improve SIC rates.^33^ By modifying the way choices are framed, nudges redirect decisions toward better outcomes while preserving autonomy and choice.^34,35,36,37^ Prior research has demonstrated the effectiveness of a clinician nudge combatting optimism bias and identifying patients with cancer at high risk of mortality based on a validated machine learning prognostic algorithm.^38^ This strategy, which notified clinicians before encounters with high-risk patients, led to a near 4-fold increase in SIC documentation (from 3.4% to 13.5%), resulting in routine use across our large academic cancer center.^17^ Given its effectiveness, this strategy was maintained as an active control in the present trial, which sought to evaluate scalable strategies to encourage SICs further.

The objective of this study was to test the independent and combined effects of clinician and patient nudges to increase SIC completion. We hypothesized that clinician and patient nudges together would be the most effective approach compared with an active control.^35,39,40^

## Methods

### Study Design

We conducted a 2 × 2 factorial, cluster-randomized clinical trial (NCT04867850) from September 7, 2021, to March 11, 2022, to test the effect of nudges to clinicians, patients, or both, compared with active control, on rates of SIC completion (eTable 2 in Supplement 2). This trial followed the Consolidated Standards of Reporting Trials (CONSORT) reporting guideline. The trial protocol^41^ (Supplement 1) was approved by the University of Pennsylvania institutional review board, which granted a waiver of requirements for informed consent because interventions posed minimal risk to participants.

### Participants and Setting

Eligible clinicians included medical and gynecologic oncologists and advanced practice professionals (APPs) in the outpatient setting caring for patients with solid, hematologic, or gynecologic malignant neoplasms. Participating sites included 4 hospitals and 6 community practices within the Abramson Cancer Center, which shared a common electronic medical record (EMR).^42^ In 2021, these entities served more than 16 000 new patients with cancer (53.6% female, 3.0% Hispanic, 15.6% Black). Clinicians providing exclusively survivorship, genetics, benign hematology, leukemia, or bone marrow transplant care were excluded (19 of 186 [10.2%]) due to few high-risk patients and/or suboptimal algorithm performance for these populations.^43^ Four of 167 eligible clinicians (2.4%) opted not to participate. All other eligible clinicians participated; there was no clinician attrition. All eligible clinicians completed an SIC training program as is customary at our institution.

**Figure 1.**
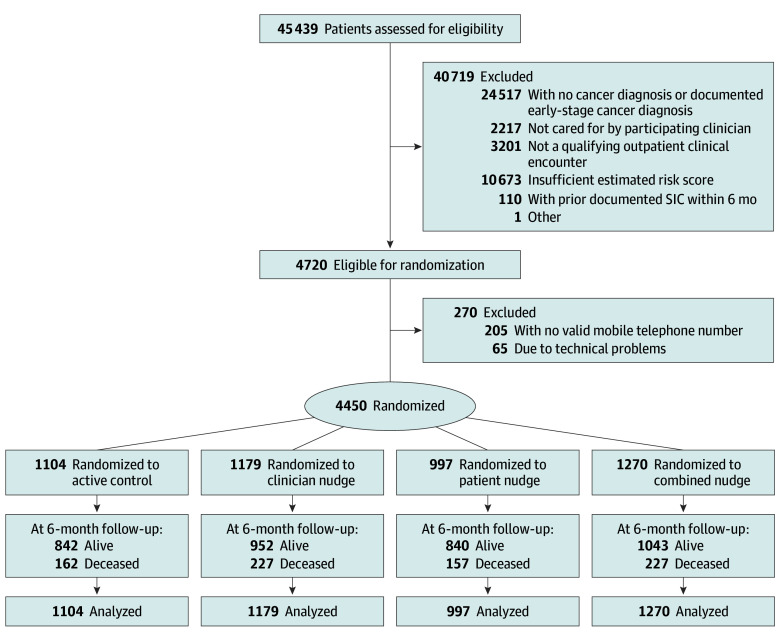
CONSORT Diagram CONSORT indicates Consolidated Standards of Reporting Trials; SIC, serious illness conversation.

Eligible patients included those receiving care from an eligible clinician, with 10% or greater estimated 180-day mortality risk based on a validated machine learning algorithm^43^ (eMethods 1 in Supplement 2) at a scheduled outpatient encounter during the study period. We excluded patients with a nonvalid mobile phone number (205 of 4720 [4.3%]) or a documented SIC within 6 months of an otherwise potentially qualifying clinical encounter (110 of 4830 [2.3%]) (Figure 1). eTable 3 in Supplement 2 details the full inclusion and exclusion criteria.

### Randomization

Randomization occurred independently at the clinician and patient levels. The clinician sample was organized into oncologist-APP clusters, which served as the unit of clinician randomization. Each oncologist-APP cluster represented an existing clinical team providing patient care together. The rationale for cluster randomization at this level was that practice patterns within teams (ie, clusters) were likely to be interdependent and highly correlated with respect to the primary outcome. Moreover, study nudges were designed to change the clinical team’s behavior, given shared responsibility for documenting SICs. Sixty-six oncologist-APP clusters were independently randomized by small-block permutation to receive the clinician nudge vs not. The patient sample consisted of eligible patients who accrued at the time of a qualifying clinical encounter, as described. Patients were independently randomized to receive the patient nudge vs not. Although study principal investigators were blinded to randomized assignment, blinding clinicians and patients was infeasible given the need to receive peer comparisons and respond to surveys, respectively.

### Implementation Strategies

Under a 2 × 2 factorial design, the independent clinician and patient randomizations yielded 4 independent study arms: (1) active control, consisting of clinician text message reminders to complete SICs for patients at high mortality risk; (2) clinician nudge only, consisting of active control plus weekly peer comparisons of clinician-level SIC completion rates (eFigure 1 in Supplement 2); (3) patient nudge only, consisting of active control plus a preclinic electronic communication designed to prime patients for SICs (eFigure 2 in Supplement 2); and (4) combined clinician and patient nudges (eTable 4 in Supplement 2). Before trial launch, we used rapid cycle approaches^44^ (eMethods 2 in Supplement 2) to optimize implementation strategy design.

### Measures

The primary outcome was SIC documentation, measured as the presence of a structured SIC smartform in a dedicated advance care planning note during a patient’s 6-month follow-up period. Documentation of SICs served as a surrogate for completed SICs, per the previous study,^17^ and is an important outcome itself, as documentation facilitates centralized communication of patient goals to care teams.^34^

Secondary outcomes were outpatient palliative care referral (which may be prompted by earlier SICs^45^) and, among decedents, aggressive end-of-life care (composite of any 1 of the following: chemotherapy within 14 days before death, hospitalization within 30 days before death, or admission to hospice within 3 days before death).^46^ eTable 5 in Supplement 2 details all outcomes. Covariates included participant age, sex, race and ethnicity, marital status, and cancer type, all of which were ascertained by EMR query. Race and ethnicity were patient reported and were assessed due to well-documented differences in end-of-life care by race and ethnicity.^47^

Sensitivity analyses ensured robustness of variable definitions. First, we expanded our primary outcome to include any structured SIC smartform in any progress note (including those documented outside of advance care planning notes). Second, we expanded the definition of aggressive end-of-life therapy to include chemotherapy or immunotherapy (rather than chemotherapy only) within 14 days before death.

### Sample Size and Power

In a priori power calculations,^41^ we sought to detect the main effects of nudges and their interaction with at least 80% power using a 2-sided α of .05. We anticipated following up with 66 oncologist-APP clusters with approximately 70 to 90 high-risk patients per cluster over the 6-month study period. Using our most conservative assumptions for patient enrollment and intraclass correlation (which was higher than intraclass correlations observed in previous SIC-focused randomized clinical trials^17^), we estimated 80% power to detect a hazard ratio (HR) of effect of 2.0 for the clinician nudge, HR of 1.3 for the patient nudge, and ratio of HRs (rHR) of 1.8 for the interaction (eTable 6 in Supplement 2). We justified these effect sizes because, in a previous trial, the adjusted effect size of a clinician-only nudge above baseline (usual care) was 2.72, and we wished to discount this because the comparator was now an active control.^17^

### Statistical Analysis

We performed an intent-to-treat (ITT) analysis at the patient level. Patients were assigned to 1 of 4 study arms according to their own randomization and that of their oncologist-APP clinical team. The primary outcome (SIC documentation) was modeled in a time-to-event analysis using a Cox proportional hazards regression model, with robust SEs accounting for patient clustering within oncologist-APP clusters (ie, the unit of clinician randomization). A single Cox proportional hazards regression model estimated HRs for the effects of clinician and patient nudges and an rHR for their interaction. The interaction was the primary estimand of interest; therefore, there was no adjustment for multiple hypothesis testing. All enrolled patients were included in the ITT analysis, and patients were censored at the time of last structured EMR activity or death, if not observed to have had a documented SIC. The model was split at 50 days, given that observed differences between arms occurred during this period. As prespecified, covariates were assessed across study arms and included in the Cox proportional hazards regression model if unbalanced (ie, statistically significantly different) across arms (age, sex). The rationale for this adjustment stemmed from the possibility that randomization of clinician clusters of varying sizes and case mixes might lead to persistent imbalances of covariates across arms.

We used the fitted Cox proportional hazards regression model to generate estimated probabilities of SIC documentation within 6 months via marginal standardization. The proportional hazards assumption was verified using scaled Schoenfeld residuals. We explored heterogeneity of effects on SIC documentation by including an interaction term between study arm and, separately, preselected patient factors (race, age, marital status). Secondary outcomes were modeled via logistic regression. Statistical significance was set at 2-sided α of .05. All analyses were conducted using Stata, version 17 (StataCorp LLC).

## Results

### Study Sample

From September 7, 2021, to March 11, 2022, the study accrued 4450 patients (median age, 67 years [IQR, 59-75 years]; 2352 women [52.9%] and 2098 men [47.1%]; 770 Black patients [17.3%], 122 Hispanic patients [2.7%], and 3294 White patients [74.0%]) seen by 163 clinicians organized into 66 oncologist-APP clusters (Table 1). Independent randomization of patients and clinician clusters yielded 4 study arms: active control (n = 1004), clinician nudge (n = 1179), patient nudge (n = 997), and combined (n = 1270). There was a slight imbalance in age and sex across arms, but no meaningful differences across arms in other covariates. Eight patients (0.2%) elected to withdraw from the study after randomization.

**Table 1.** Baseline Patient Characteristics of the Study Sample

Patient characteristic	Study arm: nudge exposure			
	Active control (n = 1004)	Clinician only (n = 1179)	Patient only (n = 997)	Both clinician and patient (n = 1270)
Age, median (IQR), y	66 (58-74)	68 (59-75)	67 (58-74)	68 (59-75)
Sex, No. (%)				
Female	556 (55.4)	594 (50.4)	558 (56.0)	644 (50.7)
Male	448 (44.6)	585 (49.6)	439 (44.0)	626 (49.3)
Race, No. (%)				
Black or African American	192 (19.1)	206 (17.5)	176 (17.7)	196 (15.4)
White	736 (73.3)	873 (74.0)	722 (72.4)	963 (75.8)
Other^a^	76 (7.6)	100 (8.5)	99 (9.9)	111 (8.7)
Ethnicity, No. (%)				
Hispanic or Latino	29 (2.9)	29 (2.5)	28 (2.8)	36 (2.8)
Not Hispanic or Latino	975 (97.1)	1150 (97.5)	969 (97.2)	1234 (97.2)
Estimated 180-d mortality, median (IQR)	0.18 (0.13-0.30)	0.19 (0.13-0.33)	0.18 (0.13-0.32)	0.19 (0.13-0.33)

^a^ “Other” included patients who identified as American Indian or Alaska Native, Asian, Native Hawaiian or Other Pacific Islander, those who identified with multiple races, and those whose racial status was unknown.

### Primary Outcome

**Figure 2.**
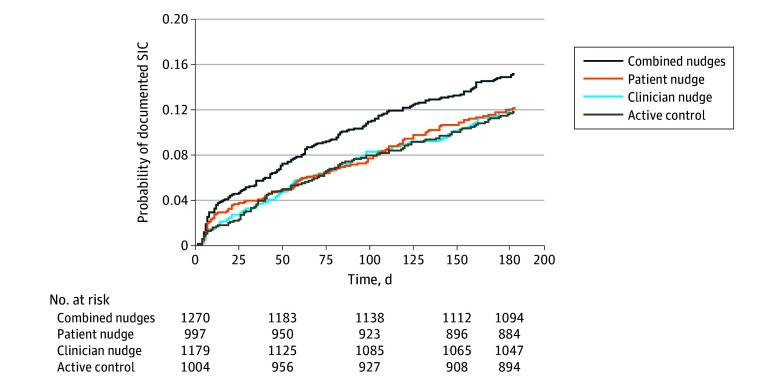
Cumulative Incidence of Serious Illness Conversations (SICs) at 6 Months by Study Arm The Nelson-Aalen estimate shows that an increased probability of a documented SIC was associated with patients receiving the patient nudge and clinicians receiving the clinician nudge.

Overall rates of 6-month SIC completion were: 11.2% for the active control group (112 of 1004), 11.5% for the clinician nudge group (136 of 1179), 11.5% for the patient nudge group (115 of 997), and 14.1% for the combined group (179 of 1270). Serious illness conversation rates diverged early for patients receiving the combined nudge, relative to other arms, and remained higher throughout follow-up (Figure 2). In adjusted analyses, compared with the active control, the combined nudges were associated with an increase in documented SIC rates (rHR, 1.55 [95% CI, 1.00-2.40]; *P* = .049), whereas the clinician nudge (HR, 0.95 [95% CI, 0.64-1.41]; *P* = .79) and patient nudge (HR, 0.99 [95% CI, 0.73-1.33]; *P* = .93) were not (Table 2).

**Table 2.** SIC Documentation by Study Arm (Intent to Treat)

Arm	Patients with SIC at 6 mo, % (95% CI)	Hazard ratios for SIC (95% CI) compared with control	*P* value
Active control	11.2 (9.3-13.3)	NA	NA
Clinician nudge	11.5 (9.8-13.5)	0.95 (0.64-1.41)	.79
Patient nudge	11.5 (9.6-13.7)	0.99 (0.73-1.33)	.93
Clinician and patient interaction	14.1 (12.2-16.1)	1.55 (1.00-2.40)^a^	.049

Abbreviations: NA, not applicable; SIC, serious illness conversation.

^a^ Represents interaction of clinician and patient nudges, expressed as ratio of hazard ratios.

**Figure 3.**
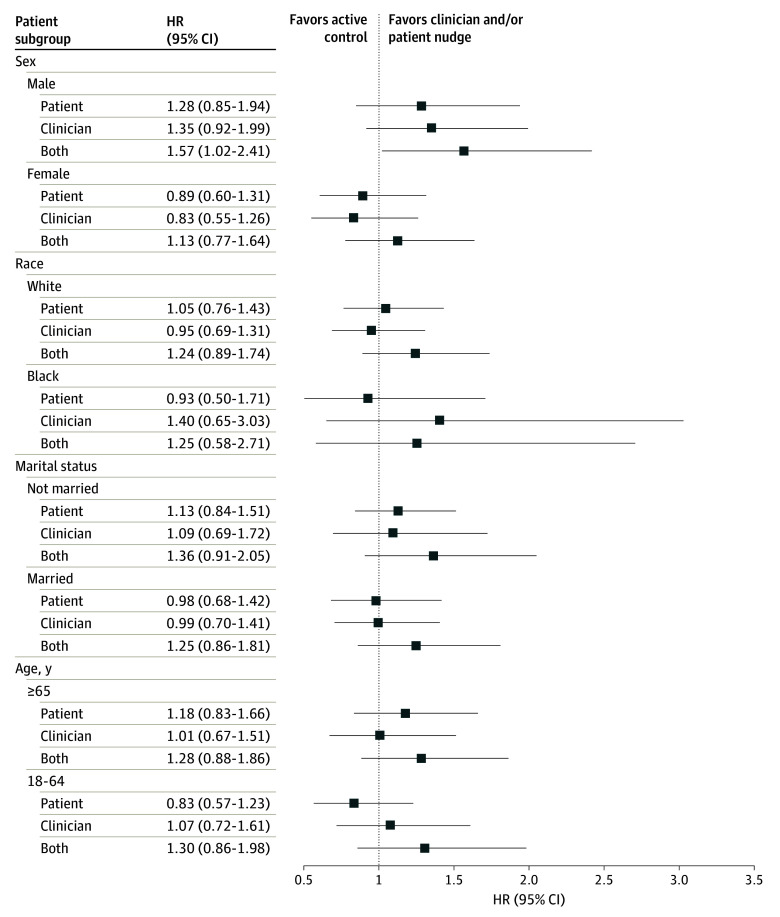
Effect Modification by Patient Subgroups Hazard ratios (HRs) represent serious illness conversation compared with active control.

In prespecified adjusted subgroup analyses comparing the combined nudge vs active control, the combined nudge was associated with higher SIC documentation for males vs females (HR, 1.57 [95% CI, 1.02-2.42] vs 1.13 [95% CI, 0.77-1.64]). However, there was no evidence of heterogeneity of effect by race, marital status, or age (Figure 3). Given small sample sizes, heterogeneity of effect for other racial groups or ethnicity was not assessed.

### Secondary Outcomes

Overall rates of 6-month palliative care referral were 8.0% in the clinician nudge arm (94 of 1179), 8.1% in the patient nudge arm (81 of 997), 7.6% in the combined arm (97 of 1270), and 8.4% in the active control arm (84 of 1004). In adjusted analyses, compared with the active control, palliative care referral rates did not differ significantly with the combined nudge (odds ratio [OR], 0.93 [95% CI, 0.68-1.26]), clinician nudge (OR, 0.98 [95% CI, 0.72-1.34]), or patient nudge (OR, 0.98 [95% CI, 0.71-1.35]) (eTable 7 in Supplement 2).

Among 773 decedents (17.4%), rates of aggressive end-of-life care were 41.9% in the clinician nudge arm (95 of 1179), 39.5% in the patient nudge arm (62 of 997), 42.7% in the combined arm (97 of 1270), and 46.9% in the active control arm (76 of 1004). In adjusted analyses, compared with the active control, aggressive end-of-life therapy rates did not differ significantly with the combined nudge (OR, 1.47 [95% CI, 0.82-2.65]), clinician nudge (OR, 0.79 [95% CI, 0.52-1.19]), or patient nudge (OR, 0.73 [95% CI, 0.47-1.15]) (eTable 8 in Supplement 2). Sensitivity analyses using broader specifications for the primary outcome and aggressive end-of-life care were consistent with the main analyses (eTable 9 in Supplement 2).

### Patient Nudge Responses

Of 2267 patients randomized to the patient nudge, the response rate was 51.4% (n = 1166). A total of 1260 patients (55.6%) viewed the priming questionnaire, 56 (2.5%) unsubscribed, and 8 (0.4%) reported concerns. Most respondents (88.3% [1030 of 1166]) expressed a wish to know “all the details” from their care team and 62.1% (724 of 1166) were willing to go through “everything” to potentially live longer.

## Discussion

In this cluster randomized clinical trial of 4450 patients with cancer at high risk of mortality, relative to an active control, the addition of clinician nudges consisting of peer comparisons and patient nudges consisting of priming questionnaires was associated with a 2.9–percentage point increase in the rate of documented SICs, whereas neither clinician nor patient nudges alone increased SIC rates. Our active control was tested in a prior study (NCT03984773), which showed that a strategy consisting of clinician-directed identification of high-risk patients and opt-out text message reminders was associated with a 10–percentage point increase in SIC rates, compared with no nudge at all, among high-risk patients with cancer.^13,17^ Our present study adds to these findings by demonstrating further, albeit marginal, improvement in SIC rates using both clinician and patient nudges. The effect size is comparable with that achieved with more resource-intensive SIC educational efforts^19^ and yet is small. Three points are worth emphasis.

First, the combination of clinician peer comparisons and a patient priming nudge was associated with improved SIC rates compared with an active control, in contrast to clinician or patient nudges alone. This study’s 2 × 2 factorial design enabled evaluation of intervention effects with greater power than a 4-arm design, yielding insights into the interaction between clinician and patient strategies. On one level, our chief finding of potential synergy in clinician and patient strategies may be unsurprising, because it takes 2 participants to have a conversation. Previous qualitative work^48^ suggests that clinicians’ perceptions of patient unwillingness to engage in emotional conversations may impede SICs. Conversely, patient-focused studies suggest that physician unwillingness to initiate SICs—perhaps influenced by optimism bias—is a major barrier.^49^ Targeting these barriers simultaneously may account for the relative success of the combination nudge.

Second, patient priming questionnaires alone did not change SIC rates compared with active control. Prior evidence has suggested benefits of patient priming on SICs.^20,50,51,52^ One trial demonstrated that administration of patient surveys identifying individual preferences, barriers, and facilitators about end-of-life care was associated with a near doubling of patient-reported goals of care conversations.^18^ Conversely, our study tested a brief 3-item questionnaire developed with patient advisory groups and designed for large-scale implementation across varied practice settings. The success of a leaner, more scalable priming questionnaire such as ours may rely on concomitant clinician-directed strategies, whereas narrower implementations of in-depth questionnaires may succeed independently but lack scalability. Across 2267 patients randomized to patient nudges, the response rate was 51.4%—higher than typical research questionnaires^53^ and comparable with other patient-reported outcomes initiatives.^54^

Third, despite the use of clinician and patient nudges, more than 80% of high-risk patients lacked documented SICs in our study, suggesting a possible ceiling effect of nudges and a critical unmet need.^22^ Efficacy trials testing other priming interventions with more comprehensive surveys and more proactive communication guidance have had larger effects on SIC rates.^18^ Here, we chose shorter patient priming tools and EMR-based nudges because these were more likely to scale across health systems. However, drawing from prior evidence and clinical limitations of patient and clinician nudges, there may be a need for more personalized patient priming tools that provide active communication guidance in oncology. For example, a previous trial of the Jumpstart-Tips intervention—a bilateral, communication-priming intervention for patients—demonstrated increased EMR documentation of SICs from 17% to 62%.^18,19^ Although our participating clinicians had all been trained in the serious illness care program^55^ and adopted standard EMR documentation smart templates, other unmeasured cultural and institutional factors may have outsized roles in determining SIC uptake and may have limited the nudges’ effect.^56^ In addition, our intervention did not address other barriers to SICs (eg, comfort starting SICs, alert fatigue). Achieving larger effect sizes and broader behavioral change may require, together with nudges, more resource-intensive strategies such as collaborations with trained lay health workers.^57^

### Limitations

Our study has several limitations. First, while we observed a modest effect within the powered range for the combined nudge, we enrolled fewer patients than expected and thus may have been underpowered to detect changes in other arms or for secondary outcomes. Second, our study was set in a single health system with robust EMR infrastructure and administrative support. Although this factor may limit generalizability, if our interventions were implemented in a system without preexisting infrastructure or initiatives to support SICs, the intervention may have a larger effect due to a lower baseline rate of SICs. In addition, our study consisted of a large patient population, spanning academic and community oncology settings, which may serve as another testament to generalizability. Third, patient covariates that may have influenced SIC completion, such as educational level and/or presence of mood disorder, were not widely available. Fourth, our primary outcome of SIC documentation is not a robust measure of SIC quality or patient goal-concordant care. Although SIC documentation has been an outcome in several supportive care studies,^12,17^ future studies should assess measures of SIC quality or comprehensiveness, patient-reported outcomes, and other patient-centered outcomes as their primary outcome, as these are likely more robust success metrics of strategies to promote SICs.

## Conclusions

In this cluster randomized clinical trial of 4450 patients with cancer at high risk of mortality, nudges combining clinician peer comparisons with patient priming questionnaires were associated with a marginal increase in documented SICs compared with an active control; neither clinician nor patient nudges alone improved SIC rates. Our study may encourage future implementation strategies to improve goals of care documentation and patient-clinician communication more broadly.

## Notes

Supplement 1.
Trial Protocol

Supplement 2. **eTable 1.** Examples of Randomized Trials Aiming to Promote Serious Illness Conversations (SICs) Among Oncology Patients **eTable 2.** Randomized 2x2 Factorial Design Yielding 4 Independent Study Arms **eTable 3.** Participant Inclusion and Exclusion Criteria **eTable 4.** Description of Implementation Strategies **eTable 5.** Primary and Secondary Outcomes **eTable 6.** Power Simulations **eTable 7.** Palliative Care Referral by Study Arm **eTable 8.** Aggressive End-of-Life Care by Study Arm **eTable 9.** Sensitivity Analyses **eFigure 1.** Clinician Nudge **eFigure 2.** Patient Nudge **eMethods 1.** Description of the Machine Learning (ML) Algorithm **eMethods 2.** Rapid Cycle Approaches
eReferences

Supplement 3.
Data Sharing Statement
