# Supplementary material for: Clinician- and Patient-Directed Communication Strategies for Patients With Cancer at High Mortality Risk: A Cluster Randomized Trial
Source: JAMA Netw Open. 2024 Jul 1;7(7):e2418639. doi: 10.1001/jamanetworkopen.2024.18639 (PMC11217875; doi:10.1001/jamanetworkopen.2024.18639)
Supplement: Supplement 2. — eTable 1. Examples of Randomized Trials Aiming to Promote Serious Illness Conversations (SICs) Among Oncology Patients eTable 2. Randomized 2x2 Factorial Design Yielding 4 Independent Study Arms eTable 3. Participant Inclusion and Exclusion Criteria eTable 4. Description of Implementation Strategies eTable 5. Primary and Secondary Outcomes eTable 6. Power Simulations eTable 7. Palliative Care Referral by Study Arm eTable 8. Aggressive End-of-Life Care by Study Arm eTable 9. Sensitivity Analyses eFigure 1. Clinician Nudge eFigure 2. Patient Nudge eMethods 1. Description of the Machine Learning (ML) Algorithm eMethods 2. Rapid Cycle Approaches eReferences [file jamanetwopen-e2418639-s002.pdf]

Supplementary Online Content

Takvorian SU, Gabriel P, Wileyto EP, et al. Clinician- and patient-directed communication strategies for patients with cancer at high mortality risk: a cluster randomized trial. *JAMA Netw Open.* 2024;7(7):e2418639. doi:10.1001/jamanetworkopen.2024.18639

eTable 1. Examples of Randomized Trials Aiming to Promote Serious Illness Conversations (SICs) Among Oncology Patients

eTable 2. Randomized 2x2 Factorial Design Yielding 4 Independent Study Arms

eTable 3. Participant Inclusion and Exclusion Criteria

eTable 4. Description of Implementation Strategies

eTable 5. Primary and Secondary Outcomes

eTable 6. Power Simulations

eTable 7. Palliative Care Referral by Study Arm

eTable 8. Aggressive End-of-Life Care by Study Arm

eTable 9. Sensitivity Analyses

eFigure 1. Clinician Nudge

eFigure 2. Patient Nudge

eMethods 1. Description of the Machine Learning (ML) Algorithm

eMethods 2. Rapid Cycle Approaches

eReferences.

This supplementary material has been provided by the authors to give readers additional information about their work.

© 2024 Takvorian SU et al. *JAMA Network Open.*

eTable 1. Examples of Randomized Trials Aiming to Promote Serious Illness Conversations (SICs) Among Oncology Patients

**Trial**	**SIC interventions**	**Impact**
Manz et al., 2020[1]	Identify patients with high mortality risk using a validated machine-learning prognostic algorithm and notify clinicians prior to encounters	Increase in SIC rates from 3.4% to 13.5%
Curtis et al., 2018[2]	Patients were prompted to complete surveys related to end-of-life care. Responses supported the population of a JumpStart guide sent to both patients and clinicians, which included barriers and facilitators and communication tips.	Increase in rates of patient-reported goals of care conversations from 31% to 74%
Bernacki et al., 2019[3]	For clinicians: Serious Illness Conversation Guide, interactive training<br>For patients and families: Letter highlighting the conversation guide, Family Guide for post-SIC discussions<br>System changes: mortality risk predictions, email reminders for clinicians, new template for SIC documentation	No significant differences in goal-concordant care, decrease in patient anxiety and depression
Oo et al., 2023[4]	Development and validation of a machine-learning algorithm to generate mortality risk predictions; clinicians are automatically alerted for intermediate- and high-risk patients	SIC rate approximately doubled
Lee et al., 2022[5]	Patients were provided "Jumpstart" guides, which included EHR data and were intended to prompt goals of care discussions	No significant differences in patient-reported discussion quantity or quality; documentation was higher in intervention arm (8% vs. 21%)
Curtis et al., 2023[6]	Clinicians received one-page "Jumpstart" guides tailored to each patient in order to prime goals of care discussions	Increase in SIC rates from 30.4% to 34.5%

© 2024 Takvorian SU et al. *JAMA Network Open.*

eTable 2. Randomized 2x2 Factorial Design Yielding 4 Independent Study Arms

		Patient: *No nudge*	Patient: *Nudge*
**Clinician**	*No Nudge*	**Usual care (active control)** *Identification of high-risk patients + performance feedback without clinician peer comparison*	**Nudge to patient only** *Normalizing message + patient priming questionnaire*
**Clinician**	*Nudge*	**Nudge to clinician only** *Identification of high-risk patients + performance feedback with clinician peer comparison*	**Nudge to clinician & patient** *Strategies from Arms 1 and 2 in combination*

© 2024 Takvorian SU et al. *JAMA Network Open.*

eTable 3. Participant Inclusion and Exclusion Criteria

**Clinicians**	
Inclusion	*Exclusion*
Medical oncologists, gynecologic oncologists, and advanced practice providers (APPs, i.e. physician assistants, nurse practitioners)	Provide exclusively survivorship, genetics, benign hematology, leukemia, or bone marrow transplant care
Provide care at least 1 clinic session per week for adult (age>18 years) patients with solid, hematologic, or gynecologic malignancies at a participating Penn Medicine practice site	
**Patients**	
Inclusion	*Exclusion*
Receive care for a solid, hematologic, or gynecologic malignancy from an eligible clinician at a participating Penn Medicine practice site	No cancer diagnosis or documented early-stage cancer diagnosis
Have at least one scheduled outpatient clinical encounter (either in person or via telemedicine) during study period	Not cared for by participating clinician
	Not a qualifying outpatient clinical encounter
	Insufficient predicted risk score
	Have a previously documented SIC within 6 months of enrollment
	Have a non-valid mobile phone number

© 2024 Takvorian SU et al. *JAMA Network Open.*

eTable 4. Description of Implementation Strategies

(i) **Active control**, consisting of identification of high-risk patients and opt-out text message reminders on clinic days to complete SICs. This approach has been described in detail previously.[1] By reminding clinicians of patients' mortality risk, this nudge aims to mitigate optimism bias and encourage clinicians to not delay SICs. All text messages were sent to work-sponsored cell phones compliant with confidentiality regulations.

(ii) **Clinician nudge only**, consisting of the active control *plus* peer comparisons on clinician-level SIC completion rates. Each week, clinicians received an email comparing their performance on SIC documentation over the previous 4 weeks to peers at their practice site and, when applicable, within their disease group (e.g., breast, colorectal, lung) (**eFigure 1**). If clinicians see their peers engaging in more SICs, they may want to fit in with their peers and engage in more SICs in the future.

(iii) **Patient nudge only**, consisting of the active control *plus* electronic patient-directed outreach designed to "prime" patients for SICs with their clinical team. Priming is a type of nudge that frames information to activate a patient's self-efficacy and willingness to engage in behavior change[7] and has the potential to improve SIC engagement.[2,5,8] By noting that the survey questions start the conversation and helping patients plan for additional discussion, the nudge seeks to overcome barriers tied to patient concerns about initiating an SIC.

Sent via text message and email before a clinical encounter, the patient priming nudge consisted of a normalizing message prompting patients to complete a brief electronic questionnaire related to their values and care preferences. Its content stemmed from a patient-facing tool developed by Ariadne Labs,[9] modified with input from focus groups with patients and caregivers. Messaging was operationalized via Penn Way to Health, an evidence-based patient engagement platform,[10,11] and integrated into the EMR. Patient-reported data from the priming questionnaire were shared with clinical teams in real-time via the EMR (**eFigure 2**). Patients had the opportunity to opt out of receiving text message and/or email communications.

(iv) **Combined clinician and patient nudges**, consisting of both clinician and patient implementation strategies described above.

© 2024 Takvorian SU et al. *JAMA Network Open.*

eTable 5. Primary and Secondary Outcomes

Primary Outcome	***Patient Population***	***Description***	***Ascertainment***
SIC documentation	High-risk patients	Measured at patient level as binary outcome, defined by presence of a documented note within the electronic medical record (EMR) using a standardized SIC template	6-month period following index clinical encounter
Secondary Outcomes	***Patient Population***	***Description***	***Ascertainment***
Palliative care referral	High-risk patients	Measured at patient level as binary outcome, defined as encounter with palliative care clinician	6-month period following index clinical encounter
Aggressive end-of-life care	High-risk decedents	Measured at patient level as a binary outcome, defined as including any of:<br>- chemotherapy within 14 days before death<br>- hospitalization within 30 days before death<br>- admission to hospice 3 days or less before death	

© 2024 Takvorian SU et al. *JAMA Network Open.*

eTable 6. Power Simulations

Patients per Cluster	Within Cluster Correlation	Detectable True Effect		
		**Clinician nudge [HR (power)]**	**Patient nudge [HR (power)]**	**Interaction [rHR (power)]**
70	0.1	1.6 (86%)	1.25 (84%)	1.6 (80%)
70	0.3	2.0 (85%)	1.3 (90%)	1.8 (91%)
90	0.1	1.6 (91%)	1.25 (86%)	1.6 (85%)
90	0.3	2.0 (86%)	1.25 (84%)	1.6 (83%)

Prior studies from our teams have had within cluster correlations of approximately 0.1.[1,12] We used the most conservative assumptions for patient enrollment and intraclass correlation (row 2 above), which relied on within cluster correlations that were higher than those found in prior studies.

© 2024 Takvorian SU et al. *JAMA Network Open.*

eTable 7. Palliative Care Referral by Study Arm

Arm	Odds Ratio of Palliative Care Referral Rate	95% Confidence Interval	P-value
**Clinician Nudge**	0.98	0.72-1.34	0.91
**Patient Nudge**	0.98	0.71-1.35	0.90
**Combined Nudge**	0.93	0.68-1.26	0.63

The active control arm is the referent group.

© 2024 Takvorian SU et al. *JAMA Network Open.*

eTable 8. Aggressive End-of-Life Care by Study Arm

Arm	Odds Ratio of Aggressive End-of-Life Care	95% Confidence Interval	P-value
**Clinician Nudge**	0.79	0.52-1.19	0.26
**Patient Nudge**	0.73	0.47-1.15	0.17
**Clinician & Patient Nudge Interaction**	1.47	0.82-2.65	0.19

The active control arm is the referent group.

© 2024 Takvorian SU et al. *JAMA Network Open.*

eTable 9. Sensitivity Analyses

Structured serious illness conversation documentation (only using the SIC template within an ACP note) by study arm (intent-to-treat)

Arm	Hazard ratio for SIC, compared to control	95% Confidence Interval	P-value
**Clincian Nudge**	0.82	0.50-1.33	0.42
**Patient Nudge**	1.12	0.76-1.64	0.57
**Clinician & Patient Nudge Interaction**	1.79	1.03-3.13	0.04

The active control arm is the referent group.

Aggressive end-of-life care (including immunotherapy) by study arm

Arm	Odds Ratio of Aggressive End-of-Life Care	95% Confidence Interval	P-value
**Clincian Nudge**	0.83	0.55-1.26	0.39
**Patient Nudge**	0.73	0.47-1.15	0.17
**Clinician & Patient Nudge Interaction**	1.39	0.78-2.50	0.27

The active control arm is the referent group.

© 2024 Takvorian SU et al. *JAMA Network Open.*

eFigure 1. Clinician Nudge

Clinician nudge: Peer comparison performance feedback, and identification of high-risk patients

Dear [clinician name]:

The ACC is working to help oncologists have earlier Serious Illness Conversations with patients.

This chart shows how many conversations you've documented in the last 3 months relative to other individuals from your disease team or practice.

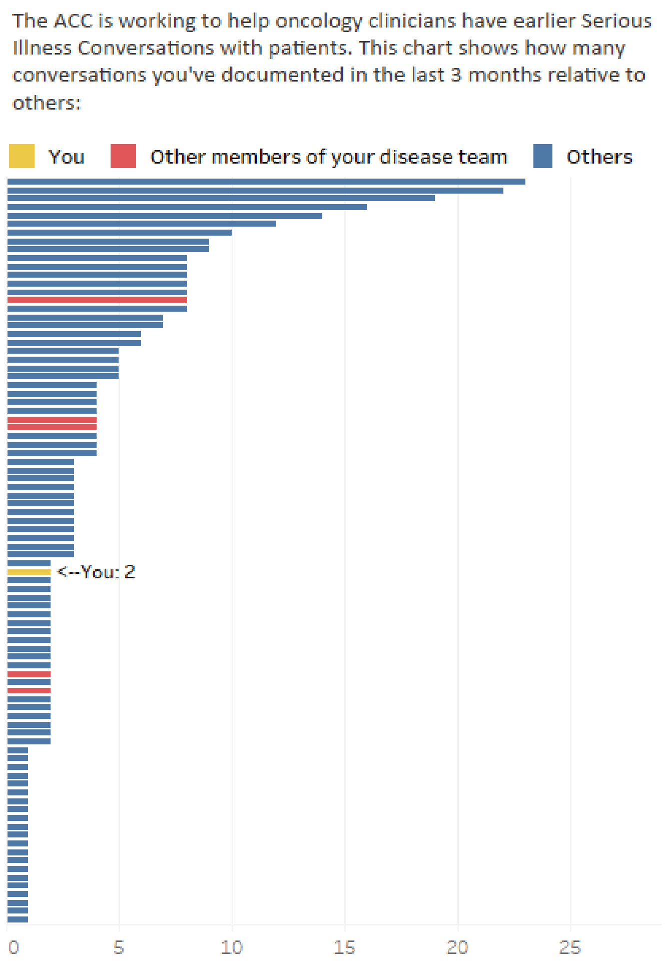

We have identified patients scheduled to see you next week who may benefit from a Serious Illness Conversation. **Click here** to view your list (you must be connected to the UPHS network).

Sincerely,

[Abramson Cancer Center Leadership]

© 2024 Takvorian SU et al. *JAMA Network Open.*

eFigure 2. Patient Nudge

Normalizing message

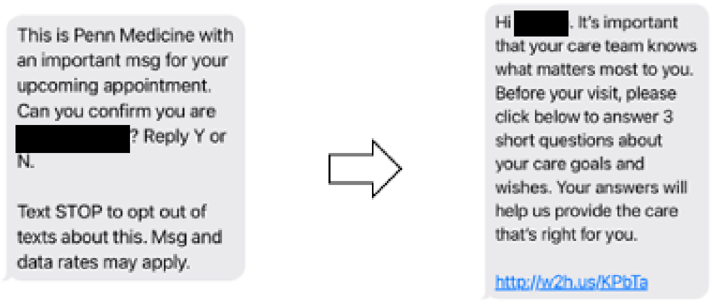

Priming questionnaire

Share what matters most to you

It's always important for your cancer care team to understand your goals and wishes, so these can be respected at every step in your cancer care. As part of a new initiative to encourage these conversations, we ask that you fill out 3 short questions, which will be shared securely with your care team. These questions were developed in collaboration with patients with cancer being treated at Penn Medicine. Questions like these can be difficult or upsetting to some people; answering them is optional and does not commit you to a particular approach. Your answers will help start the conversation between you and your cancer care team.

1. How much information about what might be ahead with your cancer would you like from your health care team?

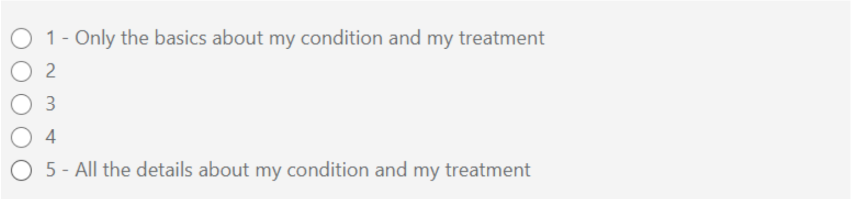

2. What is your understanding now of where you are with your cancer?

© 2024 Takvorian SU et al. *JAMA Network Open.*

3. If you become sicker, how much are you willing to go through for the possibility of living longer?

- ◯ 1 - Nothing: I wouldn't want to go through any more medical treatments
- ◯ 2
- ◯ 3
- ◯ 4
- ◯ 5 - Everything: I would want to try any medical treatments possible

© 2024 Takvorian SU et al. *JAMA Network Open.*

eMethods 1. Description of the Machine Learning (ML) Algorithm

High-risk patients were identified using a gradient-boosted ML algorithm used to predict 180-day mortality among outpatients with cancer. We previously trained this algorithm, which uses 559 structured EHR features as inputs, using historic data from 2016.[13] Of note, certain variables, including Eastern Cooperative Oncology Group (ECOG) performance status, stage, and genetic variants, were not included as features in the algorithm because they were not reliably coded in structured format at the time of algorithm training. Algorithm specifications, features, handling of missing data, cross-validation, and source code are described in this previous publication. The algorithm was integrated into a predictive engine that ran weekly on data downloaded from the Clarity database, a warehouse of data from EPIC-based electronic health vendors. Of the eligible practices in this study, 7 medical oncology practices were part of the original algorithm training data set. This algorithm was prospectively validated[14] using data in 2019 on data across the Abramson Cancer Center. The algorithm was not recalibrated after training; thus, all features and weights were locked before implementation as part of this study.

© 2024 Takvorian SU et al. *JAMA Network Open.*

eMethods 2. Rapid Cycle Approaches[15]

Rapid cycle approaches included: design meetings with subject matter experts in behavioral design (co-authors DAA and AMB), implementation science (co-authors KAR, RCS, and RSB), and health equity research (co-authors KAR, RCS, KHC, and OMF); in-depth discussions with oncology clinicians recruited from ten practice sites; and focus group discussions with members of a patient and caregiver cancer advisory group. We then engaged in multiple rounds of usability testing with clinicians and patients, which informed the design of the clinician peer comparisons and final content and mode of administration of the patient nudge.

One key change dealt with the delivery mechanism. Based on health inequities known to be associated with patient portal access and input from our informants, we shifted towards reaching out to patients via text message instead of the patient portal. Clinicians focused on the number of questions in their reviews, balancing gaining information and not being overwhelming. In addition, they recommended wording changes given the remote nature of the survey. Patient and caregiver feedback focused on the introduction to the survey. Based on their input, a clarification was added to emphasize that responses were not a commitment affecting their care and that preferences could be changed at any time. Similarly, we softened the introduction (e.g., acknowledging that questions may be upsetting for some respondents) after hearing from patients and caregivers.

© 2024 Takvorian SU et al. *JAMA Network Open.*

eReferences.

1. Manz CR, Parikh RB, Small DS, et al. Effect of Integrating Machine Learning Mortality Estimates With Behavioral Nudges to Clinicians on Serious Illness Conversations Among Patients With Cancer: A Stepped-Wedge Cluster Randomized Clinical Trial. *JAMA Oncol*. Dec 1 2020;6(12):e204759. doi:10.1001/jamaoncol.2020.4759
2. Curtis JR, Downey L, Back AL, et al. Effect of a Patient and Clinician Communication-Priming Intervention on Patient-Reported Goals-of-Care Discussions Between Patients With Serious Illness and Clinicians: A Randomized Clinical Trial. *JAMA Intern Med*. Jul 1 2018;178(7):930-940. doi:10.1001/jamainternmed.2018.2317
3. Bernacki R, Paladino J, Neville BA, et al. Effect of the Serious Illness Care Program in Outpatient Oncology: A Cluster Randomized Clinical Trial. *JAMA Intern Med*. Jun 1 2019;179(6):751-759. doi:10.1001/jamainternmed.2019.0077
4. Oo TH, Marroquin OC, McKibben J, Schell JO, Arnold RM, Kip KE. Improved Palliative Care Practices Through Machine-Learning Prediction of 90-Day Risk of Mortality Following Hospitalization. *NEJM Catalyst Innovations in Care Delivery*. 2022;4(1)doi:DOI: 10.1056/CAT.22.0214
5. Lee RY, Kross EK, Downey L, et al. Efficacy of a Communication-Priming Intervention on Documented Goals-of-Care Discussions in Hospitalized Patients With Serious Illness: A Randomized Clinical Trial. *JAMA Netw Open*. Apr 1 2022;5(4):e225088. doi:10.1001/jamanetworkopen.2022.5088
6. Curtis JR, Lee RY, Brumback LC, et al. Intervention to Promote Communication About Goals of Care for Hospitalized Patients With Serious Illness: A Randomized Clinical Trial. *JAMA*. Jun 20 2023;329(23):2028-2037. doi:10.1001/jama.2023.8812
7. Cohn A, Maréchal MA. Priming in Economics. *Current Opinion in Psychology*. 2016;12:17-21.
8. Au DH, Udris EM, Engelberg RA, et al. A randomized trial to improve communication about end-of-life care among patients with COPD. *Chest*. Mar 2012;141(3):726-735. doi:10.1378/chest.11-0362
9. Ariadne Labs. What Matters to Me Workbook. Accessed July 20, 2023, https://www.ariadnelabs.org/2021/11/16/what-matters-to-me-workbook/
10. Asch DA, Volpp KG. On the Way to Health. *LDI Issue Brief*. Jul-Aug 2012;17(9):1-4.
11. Asch DA, Muller RW, Volpp KG. Automated hovering in health care--watching over the 5000 hours. *N Engl J Med*. Jul 5 2012;367(1):1-3. doi:10.1056/NEJMp1203869
12. Jenssen BP, Schnoll R, Beidas RS, et al. Cluster Randomized Pragmatic Clinical Trial Testing Behavioral Economic Implementation Strategies to Improve Tobacco Treatment for Patients With Cancer Who Smoke. *J Clin Oncol*. Oct 1 2023;41(28):4511-4521. doi:10.1200/JCO.23.00355
13. Parikh RB, Manz C, Chivers C, et al. Machine Learning Approaches to Predict 6-Month Mortality Among Patients With Cancer. *JAMA Netw Open*. Oct 2 2019;2(10):e1915997. doi:10.1001/jamanetworkopen.2019.15997
14. Manz CR, Chen J, Liu M, et al. Validation of a Machine Learning Algorithm to Predict 180-Day Mortality for Outpatients With Cancer. *JAMA Oncol*. Nov 1 2020;6(11):1723-1730. doi:10.1001/jamaoncol.2020.4331
15. Takvorian SU, Bekelman J, Beidas RS, et al. Behavioral economic implementation strategies to improve serious illness communication between clinicians and high-risk patients with cancer: protocol for a cluster randomized pragmatic trial. *Implement Sci*. Sep 25 2021;16(1):90. doi:10.1186/s13012-021-01156-6

© 2024 Takvorian SU et al. *JAMA Network Open.*
