# Supplementary material for: Clinician- and Patient-Directed Communication Strategies for Patients With Cancer at High Mortality Risk: A Cluster Randomized Trial
Source: JAMA Netw Open. 2024 Jul 1;7(7):e2418639. doi: 10.1001/jamanetworkopen.2024.18639 (PMC11217875; doi:10.1001/jamanetworkopen.2024.18639)
Supplement: Supplement 3. — Data Sharing Statement [file jamanetwopen-e2418639-s003.pdf]

Data Sharing Statement

Takvorian. Clinician- and Patient-Directed Communication Strategies for Patients With Cancer at High Mortality Risk. *JAMA Netw Open*. Published July 01, 2024. doi:10.1001/jamanetworkopen.2024.18639

Data
Data available: No
